# Root morphology and alveolar bone phenotype of maxillary second molars in patients with periodontitis: a cross-sectional study

**DOI:** 10.1186/s12903-026-09365-5

**Published:** 2026-08-19

**Authors:** Xinyue Liu, Yifan Xie, Chuyi Jin, Mengyao Ding, Jiatong Zhang, Mengqing Yan, Lipei Liu, Ke Deng, Cheng Ding, Xing Chen

**Affiliations:** 1 Stomatology Center, Affiliated Hospital of Hangzhou Normal University, Hangzhou, 310015 Zhejiang Province China; 2 Stomatological Hospital Affiliated Suzhou Vocational Health College, Suzhou, Jiangsu Province China; 3 School of Stomatology, Hangzhou Normal University, Hangzhou, Zhejiang Province China; 4 Division of Periodontology & Implant Dentistry, Faculty of Dentistry, The University of Hong Kong, Hong Kong, SAR China

**Keywords:** Maxillary second molars, Root morphology, Periodontitis, Alveolar bone loss, Furcation involvement, Clinical attachment loss

## Abstract

**Background:**

Tooth anatomy is a key factor in periodontitis, and existing evidence has largely focused on premolars. The maxillary second molar (MSM), which is frequently affected and therapeutically challenging, remains substantially underexplored. This study aims to evaluate the root morphology and alveolar bone phenotype of the MSM in relation to periodontal parameters, aiming to identify high-risk anatomical sites and inform personalized management.

**Methods:**

This cross-sectional study included patients diagnosed with periodontitis at the Affiliated Hospital of Hangzhou Normal University between January 2022 and January 2025. Periodontal clinical parameters, including probing depth (PPD), clinical attachment loss (CAL), root furcation involvement (FI), and others, were recorded for all patients. Cone-beam computed tomography was used to assess alveolar bone loss (ABL), root concavity (angle and depth), root trunk length, root proximity, and adjacent third molars (M3s). Data were analyzed using SPSS, with *p* < 0.05 considered statistically significant.

**Results:**

The study included 319 patients (173 females and 146 males) with 576 MSMs aged 18 to 80 years (mean 43.5 ± 12.34 years). Triple-rooted (TR) morphology was most prevalent (55.21%, 318/576), followed by double-rooted (DR, 24.65%), single-rooted (SR, 19.44%), and quadruple-rooted (QR, 0.69%) morphologies. Both the angle and depth of root concavity were positively correlated with CAL and ABL. In the DR- and TR-types, inter-root proximity may serve as a potential risk indicator for FI. Thicker alveolar bone correlated with lower odds of stage III/IV and grade B/C periodontitis. The presence of adjacent M3s was associated with increased distal PPD, CAL, and ABL in MSMs.

**Conclusions:**

Our findings indicate that the specific root morphology and alveolar bone phenotype of MSMs are significantly associated with periodontitis. Anatomical features such as root concavity, inter-root proximity, and adjacent maxillary third molars may serve as potential risk indicators for localized periodontal destruction.

**Supplementary Information:**

The online version contains supplementary material available at https://doi.org/10.1186/s12903-026-09365-5.

## Background

Periodontitis is a common inflammatory disease that primarily affects the supporting tissues around the teeth, leading to gingival bleeding, the formation of periodontal pockets, and the loss of alveolar bone, ultimately resulting in tooth loosening [1]. The global prevalence of periodontitis remains relatively high and tends to increase with age [2, 3]. According to the Fourth National Oral Health Epidemiological Survey, the prevalence of periodontitis among Chinese adults older than 35 years is as high as 52.8%, with variations observed among different age groups. Notably, the highest severity is seen in the age group of 55–64 years with a prevalence of 69.3% [4].

The plaque microbiota is an important initiating factor in this process, but the pathogenesis of periodontitis is multi-dimensionally regulated including genetic susceptibility, environmental influences, and the host immune response [5, 6]. Recent studies have highlighted the important role of tooth anatomical characteristics, such as crown-root ratio, root concavity, and cervical enamel projections, in the progression of periodontitis [7, 8]. Local inflammation is significantly caused by palatal grooves and root concavities [9]. The deep and narrow palatal groove of the maxillary lateral incisor and the mesial root concavities of maxillary premolars promote plaque retention, facilitating the formation of periodontal pockets and the loss of alveolar bone [10–14].

Molar anatomy also affects periodontal health. For example, the distal-lingual root of the mandibular first molars may aggravate alveolar bone loss [15]. Root fusion is more common in second molars lost because of severe periodontitis. Additionally, the earlier exposure of radicular grooves to the oral cavity facilitates plaque accumulation, significantly increasing and the risk of periodontal tissue destruction [16]. A systematic assessment of anatomical traits is essential for accurate diagnosis and effective treatment, providing a morphological basis for tailored plaque control and preventive strategies [8, 17].

Alveolar bone resorption varies greatly between tooth positions, and alveolar bone thickness varies among populations, both influencing the progression of periodontitis [18–21]. Early studies proposed that the residual bone height of maxillary molars in periodontitis patients significantly correlates with thickening of the maxillary sinus mucosa [22, 23]. Xu et al. reported greater alveolar bone resorption in the maxillary first molars of periodontitis patients than in the second molars [19]. Moreover, Pan et al. reported significant differences in alveolar bone thickness in the maxillary anterior regions between periodontitis patients and healthy individuals, with a decreased likelihood of bone fenestration in periodontitis patients [24].

In summary, tooth anatomy is closely linked to periodontitis, and understanding periodontal phenotypic characteristics is critically important [25]. However, research on root morphology has focused mainly on maxillary anterior teeth and premolars, with limited attention given to maxillary second molars (MSMs). Nevertheless, molars are especially susceptible to periodontitis, particularly maxillary molars [26, 27], and the prevalence of tooth loss attributable to periodontitis is the greatest among these teeth [28–30]. In this study, cone beam computed tomography (CBCT) images were combined with clinical periodontal parameters to comprehensively explore the influence of the root morphology and alveolar bone characteristics of MSMs on periodontitis. Additionally, it seeks to pinpoint high-risk sites for periodontitis and inform the development of personalized prevention and treatment strategies.

## Methods

This retrospective, cross-sectional, observational study was conducted in accordance with the Declaration of Helsinki and the Strengthening the Reporting of Observational Studies in Epidemiology (STROBE) guidelines [31]. As this study involved extraction and analysis of preexisting, deidentified medical records without direct patient involvement or alteration of clinical management, informed consent was waived. The study was approved by the Ethics Committee of the Affiliated Hospital of Hangzhou Normal University (approval no. 2025(E2)-KS-002).

### Study population

Patients with periodontitis who visited the Periodontology Department at the Center of Stomatology, Affiliated Hospital of Hangzhou Normal University, between January 1 st, 2022, and January 31 st, 2025, were enrolled. All participants underwent CBCT examinations as part of the diagnostic or treatment planning process.

The inclusion criteria were as follows: (1) age ≥ 18 years; (2) diagnosis of periodontitis according to the 2017 World Workshop on the Classification of Periodontal and Peri‐Implant Diseases and Conditions [32]; (3) presence of at least one maxillary second molar; (4) no periodontal treatment within the preceding three months; and (5) CBCT imaging available, and (6) ability to complete a full periodontal examination.

Exclusion criteria included: (1) previous orthodontic treatment; (2) systemic diseases affecting periodontal health (e.g., diabetes, cardiovascular disease, hypertension); (3) heavy smokers (> 20 cigarettes/day or > 10-year history); (4) occlusal trauma involving MSMs; (5) MSMs with crown restorations, proximal caries or external cervical resorption affecting the assessment of the cemento-enamel junction (CEJ); (6) MSMs with vertical root fractures or other abnormalities; (7) poor-quality CBCT images; and (8) other factors that impeded data collection.

### Clinical periodontal parameters

The following clinical periodontal parameters were recorded for each patient: periodontal pocket depths (PPD), gingival recession (GR), gingival index (GI), plaque index (PI), bleeding on probing (BOP), tooth mobility, and furcation involvement (FI). PPD, GR, and BOP were measured at six sites per tooth: mesiobuccal (MB), mid-buccal (B), distobuccal (DB), mesiopalatal (MP), mid-palatal (P), and distopalatal (DP). Clinical attachment loss (CAL) was calculated using PPD and GR. GI and PI were scored on a scale from 0 to 3, with higher scores indicating more severe gingival inflammation and plaque accumulation, respectively [33]. All evaluation criteria align with previous published studies [10, 34]. Demographic and clinical data, including age, sex, systemic diseases, medications, smoking history, oral hygiene habits, and prior periodontal treatment, were also documented.

### Acquisition and measurement of CBCT images

All the CBCT scans were performed by senior technicians at the Radiology Department of the Affiliated Hospital of Hangzhou Normal University. CBCT images (Galileos, Sirona, Germany) were obtained using the following parameters: 85 kV tube voltage, 5 mA tube current, 100 μm voxel size, 17s exposure time, and a scanning range of 5 × 4 cm. Images were reconstructed at 0.2 mm slice thickness to balance radiation exposure with image quality and were uploaded to SIDEXIS XG software (SICAT GmbH & Co. KG, Bonn, Germany) for analysis. All measurements were performed by a single calibrated researcher. Intra-examiner reliability was assessed by re-evaluating 30 MSMs after a 1-week interval, yielding a kappa value > 0.80.

The following anatomical parameters were assessed for each MSM (Supplementary Figure S1):

1. MSM type: classification by root numbers in MSMs. Single-rooted (SR) refers to having a single root or complete fusion of multiple roots (> 75% fused). Double-rooted (DR) was defined as two roots, with subtypes based on the fusion pattern: MB-P (fusion of the mesial buccal and palatal roots), DB-P (distal buccal and palatal), and MB-DB (mesial buccal and distal buccal). Triple-rooted (TR) refers to the presence of three independent roots, whereas quadruple-rooted (QR) has four independent roots. Notably, SR and DR types are classified together as fused roots.
2. Alveolar bone loss (ABL): Measured from the cementoenamel junction (CEJ) to the alveolar bone crest at the mesial, distal, buccal, and palatal aspects, minus 2 mm (normal height defined as ≤ 2 mm from the CEJ, noted as 0).
3. Root concavity (angle and depth): For the DR, TR, and QR types, the measurement planes align with the root furcation in the axial plane, whereas for the SR type, measurements are taken 2 mm below the CEJ. The most prominent points on the axial surfaces of the root concavity were identified and connected. The root concavity angle is the angle between the concave vertex and the two innermost concave points of the roots. The depth of the root concavity is the distance from the apex of the concavity to this connecting line.
4. Alveolar bone thickness (ABT): The buccal alveolar bone thickness was measured at the midpoint of the root for both the mesial (MBT) and distal sides (DBT) as well as the palatal bone thickness at the midpoint of the palatal root (PT) [19].
5. Root furcation angle: Defined by the root bifurcation vertex and the two inner walls. If the root is highly curved, pinpointing its location is essential. The angle connecting the curvature point to the root furcation vertex is called the root furcation angle.
6. Root trunk length (RTL): The minimum vertical distance from the CEJ to the furcation [35].
7. Root proximity: the distance between the apexes of the two roots [35].

### Statistical analysis

Prior to the study, a sample size calculation was performed using G*Power 3.1 software. The analysis was based on a linear multiple regression model (fixed model, R^2^ deviation from zero) within the *F* test family. An a priori power analysis was conducted assuming a medium effect size (f^2^ = 0.15), with a significance level of 0.05, a statistical power of 0.80, and 5 predictors. The analysis indicated that a minimum sample size of 92 teeth would be required.

Statistical analyses were conducted using SPSS 26.0 (IBM, USA) and GraphPad Prism (GraphPad Software, USA). Descriptive data are shown as the mean ± standard deviation (SD). The chi-square test was used to assess the associations of MSM types with the stage and grade of periodontitis, GI, PI, and BOP. A t test was used to analyze the relationships between root concavity, MSM types and clinical periodontal parameters. One-way ANOVA was used to compare means among more than two normally distributed variables. Regression models were used to examine the correlations between root concavity (angle/depth) and periodontal parameters. Multiple linear regression was used to assess the relationship among RTL, root proximity, root furcation angle and FI. A *p-*value of < 0.05 was considered statistically significant.

SR-, DR-, and TR-type MSMs were interpreted as primary findings, while QR-type findings were considered exploratory due to the limited sample size.

## Results

### Characteristics of the samples

**Fig. 1:**
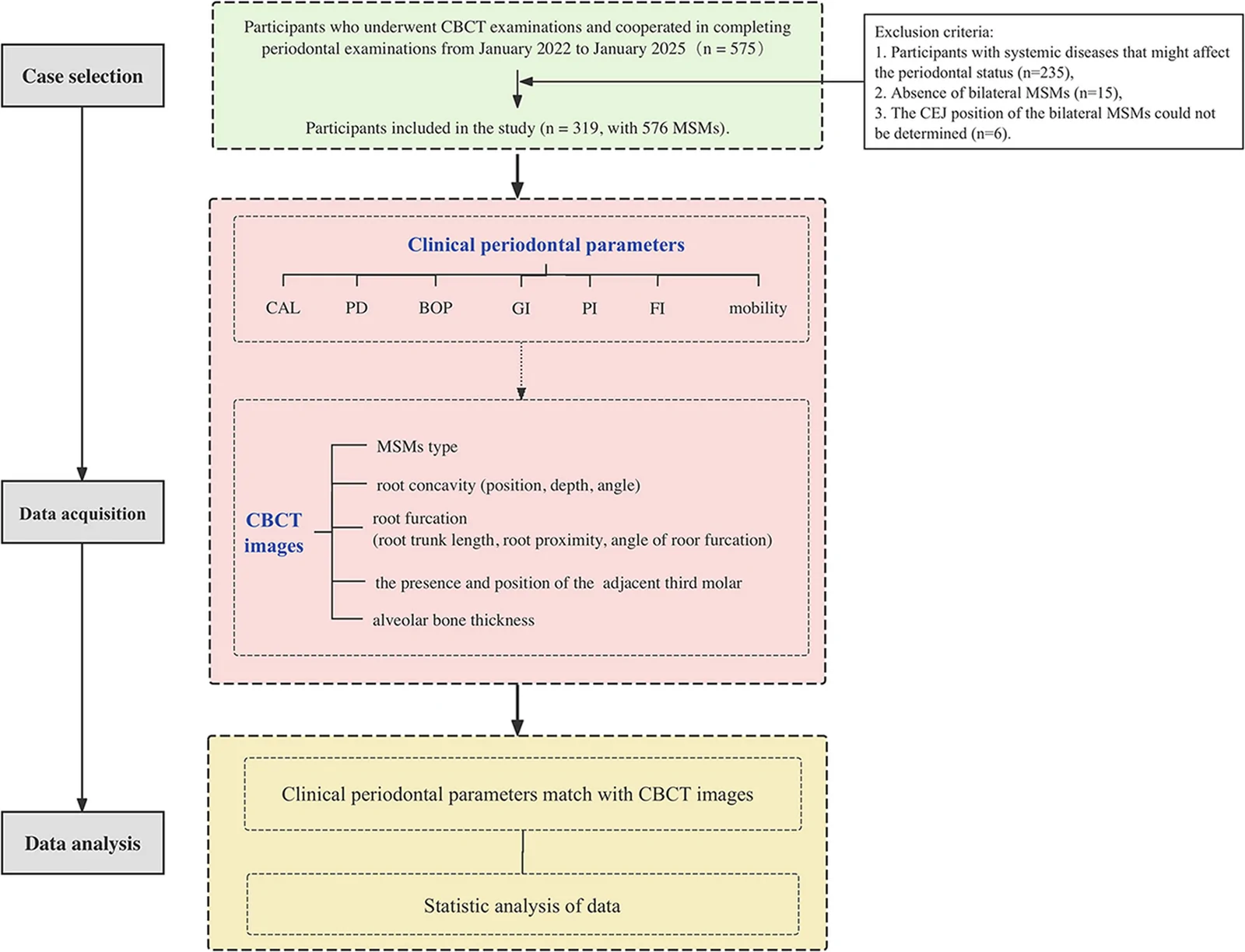
Flowchart of the patient selection and study design

**Fig. 2:**
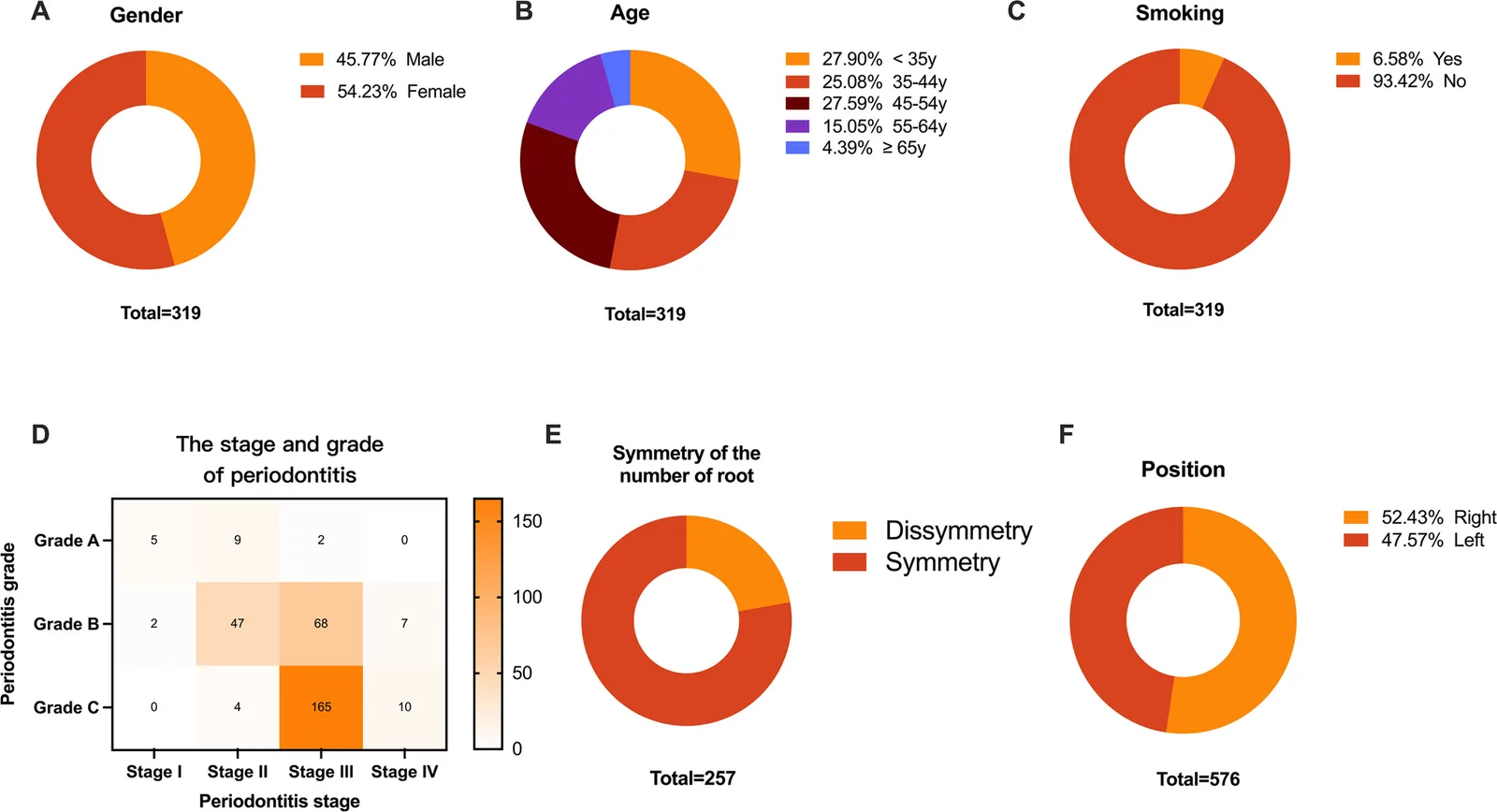
Epidemiologic characteristics of 319 patients with periodontitis

Figure 1 illustrates the process of case selection and the study design, while Fig. 2 provides an overview of patient characteristics. In total, 319 patients (146 males and 173 females) with 576 MSMs were included, with a mean age of 43.50 ± 12.34 years (range 18 to 80 years). Among the participants, 19 males and 2 females were smokers, with a male-to-female smoking ratio of approximately 11:1. Nearly 79% of patients were diagnosed with Stages III/IV periodontitis, and more than 56% had grade C periodontitis. Bilateral MSMs were present in 257 patients, while 62 had unilateral MSMs. Among those with bilateral MSMs, 77.82% exhibited the same root type bilaterally. In the 576 MSMs, the distribution was 52.43% on the right side and 47.57% on the left.

### MSM types and periodontitis

Table 1 shows the distribution of MSMs among the 576 teeth. In terms of root number, the TR type was most prevalent (55.21%, 318/576), followed by DR (24.65%, 142/576), SR (19.44%, 112/576), and QR (0.69%, 4/576). Therefore, QR-related statistical results were interpreted as exploratory and should be considered hypothesis-generating due to the small sample size. Root concavities (presence, location, and quantity) were used to further classify SRs and DRs. Among the SRs, four had no concavity, 54 had one, 29 had two, and 25 had three. The most common DR subtype was MB-P fusion, with 74 teeth, followed by MB-DB (53 teeth) and DB-P (15 teeth).

**Table 1:** Characteristics of MSMs included in the study

Variable	SR (*n* = 112)	DR (*n* = 142)	TR (*n* = 318)	QR (*n* = 4)	Total (%)
Position					
Right	58	83	158	3	302 (52.43)
Left	54	59	160	1	274 (47.57)
Numbers of root concavities					
0	4	/	/	/	4 (0.69)
1	54	/	/	/	54 (9.38)
2	29	142	/	/	171 (29.69)
3	25	/	318	/	343 (59.55)
4	0	/	/	4	4 (0.69)
Subtypes					
MB-P	/	74	/	/	74 (52.11)
DB-P	/	15	/	/	15 (10.56)
MB-DB	/	53	/	/	53 (37.32)
GI					
0	7	10	10	0	27 (4.69)
1	5	6	12	0	23 (3.99)
2	26	56	106	0	188 (32.64)
3	74	70	190	4	338 (58.68)
PI					
0	17	28	60	0	105 (18.23)
1	50	57	148	1	256 (44.44)
2	29	36	68	2	135 (23.44)
3	16	21	42	1	80 (13.89)
Mobility					
0	91	126	292	4	513 (89.06)
1	15	13	18	0	46 (7.99)
2	5	3	7	0	15 (2.60)
3	1	0	1	0	2 (0.35)
Furcation involvement					
0	/	104	213	4	321 (69.18)
≤ 1	/	23	63	0	86 (18.53)
≤ 2	/	7	24	0	31 (6.68)
≤ 3	/	8	18	0	26 (5.60)
Adjacent to the maxillary third molar					
Absent	60	70	139	4	273 (47.40)
Present	52	72	179	0	303 (52.60)

^*^SR Single-rooted, *DR* Double-rooted, *TR* Triple-rooted, *QR* Quadruple-rooted

MSM types were significantly associated with periodontitis stage (*p* < 0.01), RTL (*p* < 0.001), and root proximity (*p* < 0.001). There was also a significant difference in root number across the age groups (*p* < 0.05). No significant differences were found in terms of periodontitis grade or clinical parameters among the MSM types (*p* > 0.05). Detailed results are provided in Table 2.

**Table 2:** Clinical periodontal parameters and anatomic features by MSM root type

		**SR (*n* = 112)**	**DR (*n* = 142)**	**TR (*n* = 318)**	**QR (*n* = 4)**^**1**^	**χ2/F**	***P***
Periodontitis stage	I	2	5	7	0		
	II	23	21	65	2	27.202	< 0.01**
	III	82	107	235	0		
	IV	5	9	11	2		
Periodontitis grade	A	3	6	22	0	4.942	0.551
	B	47	51	115	1		
	C	62	85	181	3		
GI	0	7	10	10	0	15.155	0.087
	1	5	6	12	0		
	2	26	56	106	0		
	3	74	70	190	4		
PI	0	17	28	60	0	5.636	0.776
	1	50	57	148	1		
	2	29	36	68	2		
	3	16	21	42	1		
PD (Mean ± SD)		4.54 ± 1.59	4.37 ± 1.40	4.25 ± 1.25	4.42 ± 0.40	1.332	0.263
CAL (Mean ± SD)		3.75 ± 1.73	3.83 ± 1.80	3.56 ± 1.60	2.96 ± 0.58	1.202	0.308
ABL (Mean ± SD)		2.31 ± 1.70	2.42 ± 1.89	2.13 ± 1.54	1.42 ± 0.43	1.368	0.252
Age (Mean ± SD)		42.44 ± 10.95	45.44 ± 12.06	41.82 ± 12.36	41.75 ± 9.22	3.036	< 0.05*
Root trunk length (Mean ± SD)		/	5.40 ± 1.11	4.88 ± 1.14	4.53 ± 0.57	10.923	< 0.001***
Angle of root furcation (Mean ± SD)		/	40.72 ± 24.59	40.14 ± 9.71	41.74 ± 7.33	0.083	0.920
Root proximity (Mean ± SD)		/	4.82 ± 1.83	5.51 ± 1.22	4.95 ± 0.62	11.362	< 0.001***

^*^p < 0.05, **p < 0.01, ***p < 0.001

^1^ QR-type analyses were exploratory because of the very small sample size (*n* = 4); the corresponding estimates and *p* values should be interpreted with caution

### Root concavity and periodontitis

The angle of root concavity is strongly positively correlated with PD (*β* = 0.008, *p* < 0.001), CAL (*β* = 0.008, *p* < 0.001), and ABL (*β* = 0.007, *p* < 0.01). Additionally, the depth of concavity had a significant effect on CAL (*β* = 0.261, *p* < 0.01) and ABL (*β* = 0.261, *p* < 0.01). These findings indicate that larger concavity angles and deeper depths are associated with more severe periodontal destruction (Table 3).

**Table 3:** Multiple linear regression analysis of clinical parameters and root concavity among MSMs

		B	Standardized B	*P*
PD	Constant	3.272		< 0.001***
	Angle	0.008	0.128	< 0.001***
	Depth	0.147	0.061	0.068
CAL	Constant	2.251		< 0.001***
	Angle	0.008	0.125	< 0.001***
	Depth	0.261	0.099	< 0.01**
ABL	Constant	0.544		0.065
	Angle	0.007	0.116	< 0.01**
	Depth	0.261	0.104	< 0.01**

^*^p < 0.05, **p < 0.01, ***p < 0.001

Subgroup analysis by concavity position revealed that the mesial concavity angle was positively correlated with CAL (*β* = 0.009, *p* < 0.05) and ABL (*β* = 0.009, *p* < 0.05), whereas the distal concavity angle affected PD (*β* = 0.009, *p* < 0.05). The depth of the distal concavity was positively correlated with PD (*β* = 0.368; *p* < 0.01), CAL (*β* = 0.583; *p* < 0.001), and ABL (*β* = 0.477; *p* < 0.01), indicating that these sites are high risk for periodontitis. No significant correlation was found for the buccal or palatal concavities (*p* > 0.05). The results can be found in Table 4.

**Table 4:** Multiple linear regression analysis of the root concavity angle/depth and the periodontal parameters of MSMs

		PD			CAL			ABL		
		B	Standardized B	*P*	B	Standardized B	*P*	B	Standardized B	*P*
Buccal	Constant	3.520		< 0.001***	2.811		< 0.001***	0.972		< 0.05*
	Angle	0.000	0.005	0.922	0.000	0.006	0.919	0.001	0.013	0.818
	Depth	0.126	0.046	0.404	0.151	0.057	0.300	0.171	0.067	0.223
Palatal	Constant	7.153		0.076	3.477		0.229	−0.519		0.656
	Angle	−0.063	−1.504	0.128	−0.008	−0.449	0.763	0.012	0.944	0.535
	Depth	0.864	1.187	0.161	−0.076	−0.255	0.861	−0.071	−0.329	0.807
Mesial	Constant	4.197		< 0.001***	2.624		< 0.001***	0.677		0.184
	Angle	0.004	0.065	0.220	0.009	0.127	< 0.05*	0.009	0.133	< 0.05*
	Depth	0.013	0.005	0.921	0.148	0.049	0.349	0.200	0.070	0.185
Distal	Constant	2.885		< 0.001***	1.725		< 0.01**	0.823		0.187
	Angle	0.009	0.162	< 0.05*	0.008	0.117	0.082	0.002	0.031	0.644
	Depth	0.368	0.192	< 0.01**	0.583	0.247	< 0.001***	0.477	0.210	< 0.01**

^*^p < 0.05, **p < 0.01, ***p < 0.001

In the SR-type MSMs, the presence of root concavities was significantly associated with the PD/ABL of MSMs (*p* < 0.05). The concavity position influenced the aforementioned parameters and significantly affected the CAL (*p* < 0.001). The number and location of root concavities were associated with the periodontitis stage (*p* < 0.05). DR subtypes were significantly different in terms of periodontitis grade (*p* < 0.05) but were not correlated with periodontal parameters. Supplementary Materials include Table S1 and Figure S2 for relevant statistics.

Multiple regression analyses further revealed type-specific associations (Tables S2–S3). With respect to the SR-type, there was a significant positive correlation between mesial concavity depth and CAL (*β* = 1.529, *p* < 0.05). Additionally, the distal concavity angle was related to CAL (*β* = 0.03,* p* < 0.01). Distal concavity depth was strongly linked to PD (*β* = 0.880, *p* < 0.01), CAL (*β* = 1.526, *p* < 0.001), and ABL (*β* = 1.019, *p* < 0.001). For DR-type, distal concavity depth had a notable effect on CAL (*β* = 1.164, *p* < 0.05). TR-type teeth displayed different trends: the mesial concavity angle was connected to PD (*β* = 0.007, *p* < 0.05), CAL (*β* = 0.011, *p* < 0.05), and ABL (*β* = 0.012, *p* < 0.05), while mesial concavity depth influenced CAL (*β* = 0.459, *p* < 0.01). In contrast to mesial morphology, TR-type distal morphology revealed relationships where the distal concavity angle was related to CAL (*β* = 0.013, *p* < 0.01) and where the distal concavity depth impacted CAL (*β* = 0.464, *p* < 0.01) and ABL (*β* = 0.357, *p* < 0.05).

To exclude the confounding effect of adjacent M3s, we analyzed distal parameters from MSMs without M3s (Table S4). The depth of the distal concavity in an SR-type is significantly positively correlated with PD (*β* = 1.186, *p* < 0.001), CAL (*β* = 1.528, *p* < 0.001) and ABL (*β* = 1.105, *p* < 0.01). Furthermore, an increase in the angle of the distal concavity aggravated CAL (*β* = 0.020, *p* < 0.05). Additionally, an increase in the depth of the distal concavity of the DR type is associated with increased CAL (*β* = 1.763, *p* < 0.01). No significant correlations were detected at the other sites.

### Root trunk length, root proximity, root furcation angle, and furcation involvement

Table S5 illustrates correlations between RTL, root furcation angle, root proximity, and FI. These correlations are limited to DR- and TR-types (SR-type lacks furcation; QR-type is excluded because of small sample size). Logistic regression revealed no significant link between root furcation angle or RTL and FI prevalence or severity. However, root proximity is partially correlated with FI prevalence and severity. For TR-type (using FI = 3 as reference), each 1 mm increase in distance between the distobuccal and palatal roots resulted in a 28.2% reduction in the prevalence of distal FI and a 33.6% reduction in grade I FI at the distal root furcation. Similar directional trends were observed in DR-type MSMs at mesial and distal sites.

### Alveolar bone thickness and local periodontal status

The results indicated significant correlations between ABT and MSM type, with DBT showing substantial variation (*p* < 0.05). Conversely, MBT and PT are unrelated to MSM type. The results are shown in Supplementary Materials Figure S3A.

Multiple linear regression showed that MBT exhibited a negative correlation with the stage (*β* = −0.055, *p* < 0.05) and grade (*β* = −0.060, *p* < 0.05) of periodontitis, and PT was negatively associated with periodontitis stage (*β* = −0.082, *p* < 0.001). This finding indicates that elevated levels of MBT and PT are associated with less severe periodontitis, suggesting that greater MBT is linked to a slower progression of periodontitis. No other significant correlations were detected (Table S6).

### Maxillary third molars and periodontal status of adjacent MSMs

Among the 576 MSMs, 303 had adjacent M3s, whereas 273 did not. M3s were classified by their presence, intraosseous depth, and positional relationship to MSMs. The results revealed that the presence of M3s was significantly associated with increased distal PPD, CAL, and ABL in adjacent MSMs (PPD and CAL, *p* < 0.001; ABL, *p* < 0.01). However, neither the intraosseous depth nor the position of M3s correlated with the distal periodontal parameters of MSMs (*p* > 0.05). The relevant results are presented in Figures S3B–S3D.

## Discussion

Periodontitis is the leading cause of tooth loss worldwide. In 2021, severe periodontitis affected approximately 1 billion people globally, with projections reaching 1.5 billion by 2025. Periodontitis has also been linked to systemic conditions including cardiovascular disease, diabetes, and Alzheimer's disease [1, 36–38]. Public health education and enhanced prevention are urgently needed given these epidemiological trends.

Although associations between root concavity and periodontitis have been noted in premolars [39], MSMs present unique challenges due to their posterior location, frequent root concavities, and complex furcation anatomy [40]. Plaque control, periodontal instrumentation, and surgical interventions are complicated by these characteristics. It is the first study to integrate multiple MSM-specific anatomical traits with clinical periodontal parameters such as root fusion patterns, concavity depth and angle, and alveolar bone thickness. CBCT-based studies have traditionally focused primarily on root canal characteristics without relating these complex anatomical features to periodontal outcomes. The purpose of our study is to fill this critical gap.

### Description of the sample

In our study of 319 patients (146 males, 173 females), sex differences were not significant, but males had a higher level of periodontitis severity. This is likely a result of a substantially higher smoking prevalence among males (11.22 times greater than females), an established risk factor for periodontitis.

As a result of including patients with stage I–II periodontitis (approximately 21% of the cohort) rather than only those who suffered moderate-to-severe periodontitis, the mean age of our cohort was slightly younger than that reported in previous epidemiological studies [3]. Only 80.6% of patients presented with bilateral MSMs, indicating substantial tooth loss. The high proportions of patients with stage III/IV (79%) and grade C (56.11%) disease underscore the significant burden of severe periodontitis in Chinese.

### Classification of MSM morphology

Studies define fused roots differently, with most using a CEJ-to-furcation distance ratio greater than 70% of the total root length [41]. The results of our study remain unaffected despite these different criteria. MSMs with fused roots accounted for 44.1%, in accordance with Zhang's findings [41]. Conventionally, fused MSMs are classified into six types (types I–VI), with types I–III representing our DR category. It is important to note, however, that this system only describes the fusion pattern and does not describe the number or position of root concavities. Due to the differential effect of concavity location on periodontal status, we refined the conventional classification system by subclassifying Types IV–VI as SRs based on the number and location of concavities. By using this methodological innovation, confounding factors are reduced and periodontal risk is more precisely assessed as a result of concavity. Figure S1A illustrates the different types of MSM. Due to the rarity of the quadruple-rooted variant, Figure S4 and Table S7 show these cases separately.

### Impact of root concavity on periodontal destruction

Our analyses revealed a strong association between root concavity and periodontal destruction. Interestingly, buccal concavities were not correlated with periodontal parameters (except in the rare QR type). This may reflect the effectiveness of routine toothbrushing in maintaining buccal surface hygiene. Conversely, it remains challenging to clean proximal surfaces, particularly mesial and distal surfaces, despite the use of adjunctive aids such as dental floss. The limited efficacy of these aids on proximal surfaces likely explains the stronger associations observed between proximal concavities and increased localized periodontal inflammation.

### Root trunk length, root proximity and Furcation Involvement

While the literature often suggests that a shorter RTL leads to earlier furcation exposure and severe FI, a shorter RTL paradoxically improves access for surgical debridement and root resection during periodontal maintenance or guided tissue regeneration. In our study, despite significant differences in RTL between DR- and TR-type MSMs, neither RTL nor furcation angle significantly influenced FI severity. This lack of correlation may stem from the anatomical complexity of MSMs relative to mandibular molars or from variations in individual biomechanical loading. Instead, inter-root proximity emerged as a critical factor. In TR types, a 1 mm increase in distobuccal–palatal root proximity was associated with a reduction in the prevalence and severity of distal FI. This inverse relationship suggests that inter-root proximity may serve as a potential risk indicator for FI in MSMs.

### Alveolar bone thickness

Significant differences in ABT were observed across MSM types and tooth sites in relation to periodontitis. This observation relates to the anatomy of maxillary molars, where mesial and distal buccal roots are short and slender, and the alveolar bone is thinner. In contrast, the palatal roots are longer and thicker, providing better support from the alveolar bone. Additionally, a significant correlation exists between MSM types and DBT, while MBT is related to the stage and grade of periodontitis, and PT is connected to the periodontitis stage. The contributing factors include that most subjects have stage III/IV periodontitis and that the ABL is relatively severe and affects the measurement. The M3s could also affect DBT measurements, leading to discrepancies.

### Impact of adjacent third molars

More than 50% of MSMs have adjacent third molars. The presence of M3s significantly accelerates periodontal tissue destruction in MSMs, resulting in increasing distal ABL, PPD, and CAL, which hastens the onset and progression of localized periodontal inflammation. This aligns well with prior research [42–45]. Notably, the position or intraosseous nature of the M3s did not independently correlate with these parameters, suggesting that the mere presence of the M3 is the primary driver of localized inflammation rather than a direct mechanical or positional effect.

### Rare anatomical variants (Quadruple-Rooted MSMs)

In this cohort, QR-type MSMs were uncommon, with a prevalence of 0.69% (4/576). While this subgroup was retained in the descriptive statistics and exploratory analyses to provide a comprehensive overview of MSM anatomical variation, the extremely small sample size considerably limits the reliability of QR-specific estimates. Therefore, QR-related statistical findings should be interpreted as hypothesis-generating rather than confirmatory. Supplementary Figure S4 and Table S7 provides a case series of the four QR-type MSMs. This rare anatomical variant requires multicenter studies or specialized case series to better understand its implications on periodontal health and endodontics.

### Strength and limitations

Additionally, this study has several limitations that must be fully acknowledged. First, owing to the inherent nature of a cross-sectional design, we can report only associations and cannot establish causality. We cannot definitively determine whether these anatomical variants are initiating risk factors for periodontitis or if they simply represent areas more vulnerable to rapid destruction once the disease is established. Consequently, tracking individual disease progression over time was not possible. Second, the microbial composition within root concavities was not examined, limiting our insights into localized pathogenic mechanisms. Third, the reasons for the absence of M3 (e.g., prophylactic extraction vs. congenital agenesis) were not recorded, and the long-term impact of M3 was unclear. This is exactly where future research efforts should be focused. Finally, although QR-type MSMs were retained in the descriptive and exploratory analyses to preserve the completeness of anatomical classification, the subgroup included only four teeth. Therefore, QR-specific regression results may be unstable and should not be regarded as confirmatory evidence. Thus, the results must be interpreted with caution.

It is clear that our study has clinical implications despite these limitations. During diagnosis and treatment planning, clinicians should carefully evaluate the anatomical characteristics of MSMs, paying special attention to proximal surfaces. Detecting adjacent M3s and debriding deep root concavities require specialized instruments. Sustained maintenance therapy is essential.

## Conclusion

Our findings suggest that the root concavity angle and depth are positively correlated with CAL and ABL, and the concavity angle is additionally associated with PD, although the effect varies across MSM types. Inter-root proximity may serve as a potential risk indicator for FI, whereas greater alveolar bone thickness may reduce the likelihood of advanced periodontitis (stage III/IV and grade B/C). Clinicians should carefully assess these anatomical features during treatment planning. Identifying high-risk regions may aid in tailoring personalized maintenance strategies to mitigate periodontitis progression.

## Supplementary Information

Supplementary Material 1. Figure S1. Schematic diagram of imaging data measurement methods.

Supplementary Material 2. Figure S2. The correlation between the fusion roots of MSMs and clinical periodontal parameters.

Supplementary Material 3. Figure S3. The correlations between the thickness of alveolar bone and the adjacent maxillary third molars and MSMs.

Supplementary Material 4. Figure S4. CBCT images illustrating the root morphology of four QR-type MSMs. Rows indicate Cases 1–4, and columns show buccal, palatal, mesial, distal, and axial views.

Supplementary Material 5. Table S1. The correlation between the fusion roots of MSMs and the stage/grade of periodontitis.

Supplementary Material 6. Table S2. Multiple linear regression analysis of clinical parameters and root concavity among different MSMs types.

Supplementary Material 7. Table S3. Binary logistic regression analysis of BOP and root concavity.

Supplementary Material 8. Table S4. Multiple linear regression analysis of the root concavity angle/depth and the distal periodontal parameters of MSMs without adjacent third molars.

Supplementary Material 9. Table S5. Multiple logistic regression analysis between the angle of root furcation, root proximity, root trunk length and FI among MSMs type.

Supplementary Material 10. Table S6. Multiple linear regression of ABT and the stage/grade of periodontitis.

Supplementary Material 11. Table S7. A case series of QR-type MSMs.

## Data Availability

All data generated or analyzed during this study are included in this published article and its supplementary information files. For additional questions, please contact the corresponding authors (Cheng Ding: cheng0710@gmail.com, or Xing Chen: 316679138@qq.com).

## Abbreviations

ABL: Alveolar bone loss
B: Buccal
BOP: Bleeding on probing
CAL: Clinical attachment loss
CBCT: Cone beam computed tomography
CEJ: Cemento-enamel junction
DB: Distobuccal
DBT: Distal buccal alveolar bone thickness
DLR: Disto-lingual root
DP: Disto palatal
DR: Double-rooted
FI: Furcation involvement
GI: Gingival index
GR: Gingival recession
M3s: Adjacent third molars
MB: Mesiobuccal
MBT: Mesial buccal alveolar bone thickness
Mm: Millimeter
MP: Mesiopalatal
MSMs: Maxillary second molars
P: Palatal
PG: Palatal groove
PI: Plaque index
PPD: Periodontal probing depth
PT: Palatal alveolar bone thickness
QR: Quadruple-rooted
RTL: Root trunk length
SR: Single-rooted
TR: Triple-rooted
