# Supplementary material for: Root morphology and alveolar bone phenotype of maxillary second molars in patients with periodontitis: a cross-sectional study
Source: BMC Oral Health. 2026 Aug 19;26:1813. doi: 10.1186/s12903-026-09365-5 (PMC13613663; doi:10.1186/s12903-026-09365-5)
Supplement: Supplementary file 1 [file 12903_2026_9365_MOESM3_ESM.pdf]

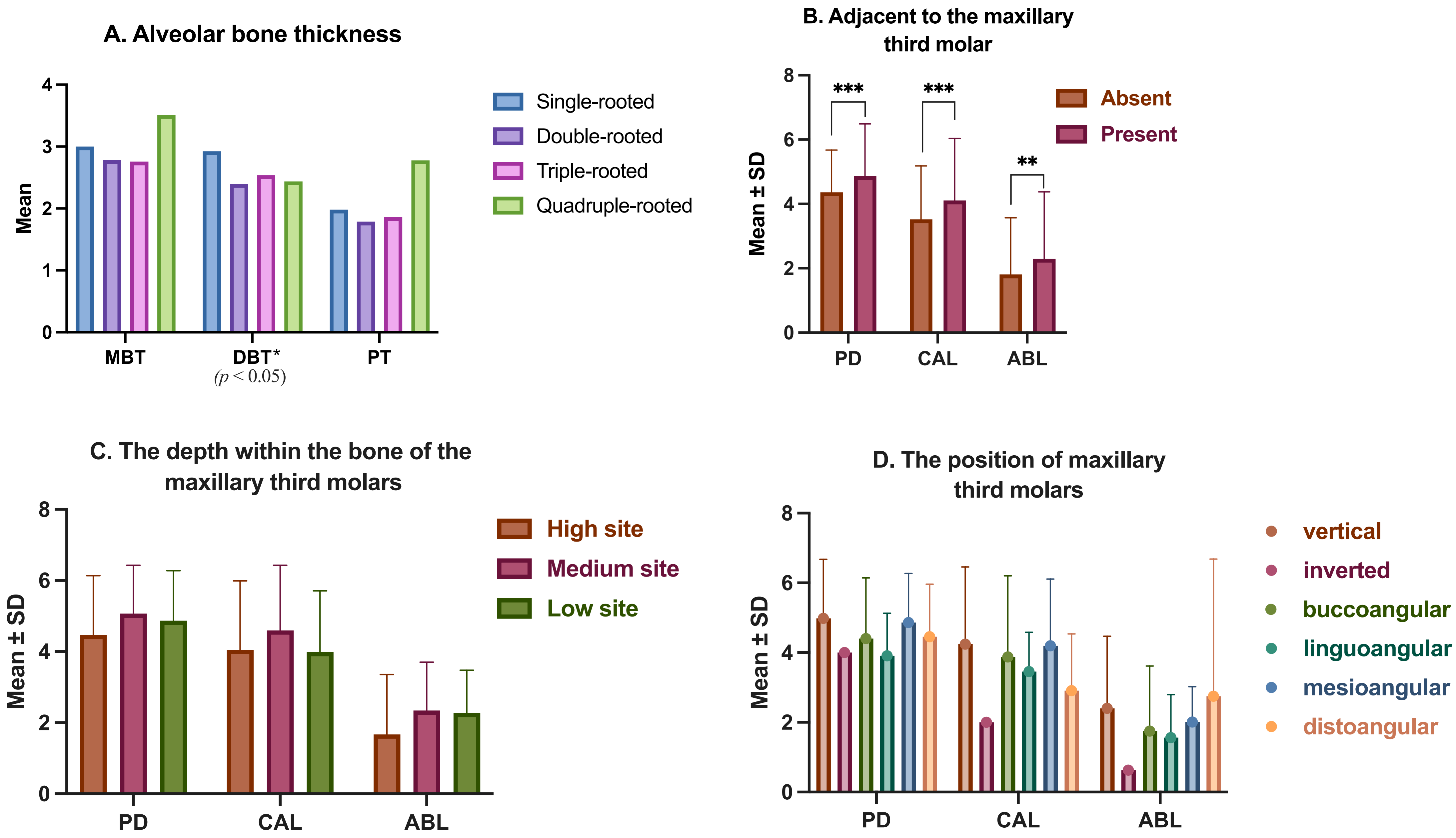

A. Alveolar bone thickness
Mean
0
1
2
3
4
MBT
DBT*
(p < 0.05)
PT
Single-rooted
Double-rooted
Triple-rooted
Quadruple-rooted
B. Adjacent to the maxillary third molar
Mean ± SD
0
2
4
6
8
***
***
**
PD
CAL
ABL
Absent
Present
C. The depth within the bone of the maxillary third molars
Mean ± SD
0
2
4
6
8
PD
CAL
ABL
High site
Medium site
Low site
D. The position of maxillary third molars
Mean ± SD
0
2
4
6
8
PD
CAL
ABL
vertical
inverted
buccoangular
linguoangular
mesioangular
distoangular
