# Supplementary material for: Root morphology and alveolar bone phenotype of maxillary second molars in patients with periodontitis: a cross-sectional study
Source: BMC Oral Health. 2026 Aug 19;26:1813. doi: 10.1186/s12903-026-09365-5 (PMC13613663; doi:10.1186/s12903-026-09365-5)
Supplement: Supplementary file 2 [file 12903_2026_9365_MOESM4_ESM.pdf]

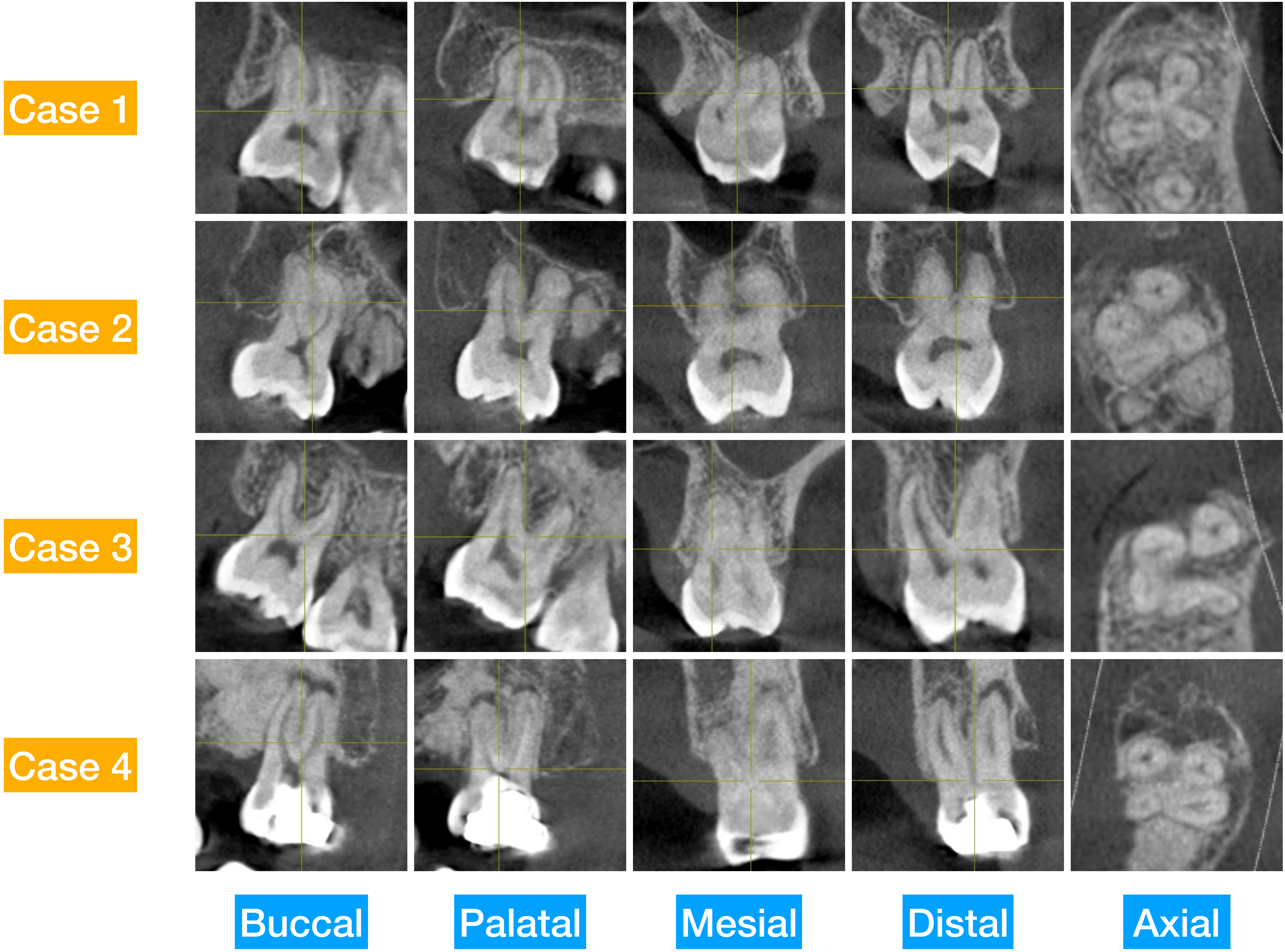

Case 1
Case 2
Case 3
Case 4
Buccal
Palatal
Mesial
Distal
Axial
