# Supplementary material for: Root morphology and alveolar bone phenotype of maxillary second molars in patients with periodontitis: a cross-sectional study
Source: BMC Oral Health. 2026 Aug 19;26:1813. doi: 10.1186/s12903-026-09365-5 (PMC13613663; doi:10.1186/s12903-026-09365-5)
Supplement: Supplementary file 3 [file 12903_2026_9365_MOESM10_ESM.docx]

Table S6. Multiple linear regression of ABT and the stage/grade of periodontitis.

|  | **Periodontitis stage** | | | **Periodontitis grade** | | |
| --- | --- | --- | --- | --- | --- | --- |
|  | B | Standardized B | *P* | B | Standardized B | *P* |
| Constant | 3.205 |  | 0 | 2.799 |  | 0 |
| MBT | -0.055 | -0.155 | **<0.05*** | -0.06 | -0.157 | **<0.05*** |
| DBT | -0.036 | -0.101 | 0.081 | -0.017 | -0.044 | 0.468 |
| PT | -0.082 | -0.178 | **<0.001***** | -0.035 | -0.07 | 0.112 |

**p*<0.05, ***p*<0.01, ****p*<0.00
