# Supplementary material for: Root morphology and alveolar bone phenotype of maxillary second molars in patients with periodontitis: a cross-sectional study
Source: BMC Oral Health. 2026 Aug 19;26:1813. doi: 10.1186/s12903-026-09365-5 (PMC13613663; doi:10.1186/s12903-026-09365-5)
Supplement: Supplementary file 4 [file 12903_2026_9365_MOESM11_ESM.docx]

Table S7. A cases series of QR-type MSMS.

| Case | Age | Sex | FDI | Periodontitis | | Clinical parameters | | | | | | | | | | | | | | | | | | | | M3 presence |
| --- | --- | --- | --- | --- | --- | --- | --- | --- | --- | --- | --- | --- | --- | --- | --- | --- | --- | --- | --- | --- | --- | --- | --- | --- | --- | --- |
|  |  |  |  | Stage | Grade | PD | | | | | | CAL | | | | | | ABL | | | | Mobility | FI | GI | PI |  |
|  |  |  |  |  |  | DB | B | MB | DP | P | MP | DB | B | MB | DP | P | MP | M | D | B | P |  |  |  |  |  |
| 1 | 45 | Female | #17 | II | B | 5 | 4 | 4 | 4 | 4 | 4 | 4 | 3 | 2 | 3 | 2 | 2 | 0.75 | 1.94 | 1.26 | 0.64 | 0 | 0 | 3 | 2 | Absence |
| 2 | 28 | Female | #17 | II | C | 4 | 3 | 5 | 4 | 3 | 6 | 2 | 2 | 2 | 4 | 2 | 2 | 0.22 | 0.96 | 0.55 | 0 | 0 | 0 | 3 | 3 | Absence |
| 3 | 47 | Male | #17 | IV | C | 5 | 3 | 4 | 8 | 6 | 4 | 4 | 3 | 3 | 4 | 3 | 5 | 0.94 | 0.20 | 3.58 | 0 | 0 | 0 | 3 | 2 | Absence |
| 4 | 47 | Male | #27 | IV | C | 6 | 4 | 5 | 4 | 3 | 4 | 4 | 4 | 3 | 2 | 3 | 3 | 1.95 | 1.41 | 1.46 | 0.79 | 0 | 0 | 3 | 1 | Absence |
| Notes: DB: distobuccal, B: buccal, MB: mesiobuccal, DP: distopalatal, P: palatal, MP: mesiopalatal, M: mesial, D: distal | | | | | | | | | | | | | | | | | | | | | | | | | | |
