# Supplementary material for: Root morphology and alveolar bone phenotype of maxillary second molars in patients with periodontitis: a cross-sectional study
Source: BMC Oral Health. 2026 Aug 19;26:1813. doi: 10.1186/s12903-026-09365-5 (PMC13613663; doi:10.1186/s12903-026-09365-5)
Supplement: Supplementary file 5 [file 12903_2026_9365_MOESM5_ESM.docx]

Table S1. The correlation between the fusion roots of MSMs and the stage/grade of periodontitis.

|  | **Single-rooted** | | | | | | | | | **Double-rooted** | | |  |
| --- | --- | --- | --- | --- | --- | --- | --- | --- | --- | --- | --- | --- | --- |
|  | **Root concavity** | | **Number of root concavities** | | | **Position of root concavities** | | | **Subtypes** | | | | |
|  | Absent | Present | 1 | 2 | 3 | Buccal | Mesial | Distal | MB-P | | DB-P | MB-DB | |
| Periodontitis stage (EFP/AAP) | | | | | | | | | | | | | |
| Stage I | 0 | 2 | 1 | 1 | 0 | 1 | 0 | 1 | 2 | | 0 | 3 | |
| Stage II | 2 | 21 | 4 | 11 | 6 | 17 | 0 | 4 | 12 | | 3 | 6 | |
| Stage III | 2 | 80 | 48 | 15 | 17 | 32 | 6 | 42 | 53 | | 10 | 44 | |
| Stage IV | 0 | 5 | 1 | 2 | 2 | 4 | 0 | 1 | 7 | | 2 | 0 | |
|  | **χ2=2.317, *p* = 0.508** | | **χ2=17.765, *p* < 0.05*** | | | **χ2 = 13.526, *p* < 0.05*** | | | **χ2=8.521, *p* = 0.202** | | | | |
| Periodontitis grade (EFP/AAP) | | | | | | | | | | | | | |
| Grade A | 0 | 3 | 0 | 3 | 0 | 3 | 0 | 0 | 1 | | 0 | 5 | |
| Grade B | 1 | 46 | 23 | 13 | 10 | 23 | 4 | 19 | 31 | | 8 | 12 | |
| Grade C | 3 | 59 | 31 | 13 | 15 | 28 | 2 | 29 | 42 | | 7 | 36 | |
|  | **χ2=0.685, *p* = 0.710** | | **χ2=8.964, *p* = 0.062** | | | **χ2=4.787, *p* = 0.310** | | | **χ2=11.218, *p* < 0.05*** | | | | |

**p*<0.05, ***p*<0.01, ****p*<0.001
