# Supplementary material for: Root morphology and alveolar bone phenotype of maxillary second molars in patients with periodontitis: a cross-sectional study
Source: BMC Oral Health. 2026 Aug 19;26:1813. doi: 10.1186/s12903-026-09365-5 (PMC13613663; doi:10.1186/s12903-026-09365-5)
Supplement: Supplementary file 6 [file 12903_2026_9365_MOESM6_ESM.docx]

Table S2. Multiple linear regression analysis of clinical parameters and root concavity among different MSMs types.

|  |  |  | **PD** | | | **CAL** | | | **ABL** | | |
| --- | --- | --- | --- | --- | --- | --- | --- | --- | --- | --- | --- |
|  |  |  | B | Standardized B | *P* | B | Standardized B | *p* | B | Standardized B | *P* |
| **SR** | Buccal | Constant | 3.196 |  | 0.034 | 3.105 |  | 0.021 | 0.812 |  | 0.449 |
|  |  | Angle | 0.005 | 0.087 | 0.627 | -0.004 | -0.075 | 0.674 | -0.002 | -0.046 | 0.797 |
|  |  | Depth | 0.095 | 0.039 | 0.828 | -0.057 | -0.026 | 0.884 | 0.134 | 0.075 | 0.676 |
|  | Mesial | Constant | 1.995 |  | 0.140 | 3.865 |  | 0.029 | 0.546 |  | 0.785 |
|  |  | Angle | 0.004 | 0.089 | 0.687 | -0.011 | -0.182 | 0.333 | -0.002 | -0.028 | 0.898 |
|  |  | Depth | 0.725 | 0.333 | 0.139 | 1.529 | 0.466 | **<0.05*** | 0.856 | 0.261 | 0.245 |
|  | Distal | Constant | 1.751 |  | 0.170 | -1.599 |  | 0.257 | -0.468 |  | 0.688 |
|  |  | Angle | 0.017 | 0.200 | 0.061 | 0.030 | 0.309 | **<0.01**** | 0.010 | 0.135 | 0.199 |
|  |  | Depth | 0.880 | 0.280 | **<0.01**** | 1.526 | 0.416 | **<0.001***** | 1.019 | 0.348 | **<0.001***** |
| **DR** | Buccal | Constant | 3.975 |  | 0.002 | 2.352 |  | 0.067 | 0.796 |  | 0.564 |
|  |  | Angle | -0.002 | -0.037 | 0.792 | 0.004 | 0.060 | 0.666 | 0.005 | 0.064 | 0.645 |
|  |  | Depth | 0.080 | 0.032 | 0.820 | 0.406 | 0.153 | 0.270 | 0.330 | 0.116 | 0.407 |
|  | Mesial | Constant | 5.054 |  | 0.000 | 4.328 |  | 0.004 | 2.093 |  | 0.055 |
|  |  | Angle | 0.005 | 0.058 | 0.641 | 0.006 | 0.063 | 0.609 | 0.005 | 0.064 | 0.605 |
|  |  | Depth | -0.438 | -0.152 | 0.223 | -0.509 | -0.149 | 0.230 | -0.317 | -0.125 | 0.315 |
|  | Distal | Constant | 3.196 |  | 0.003 | 1.056 |  | 0.463 | 0.491 |  | 0.731 |
|  |  | Angle | 0.009 | 0.137 | 0.180 | 0.010 | 0.116 | 0.250 | 0.006 | 0.063 | 0.532 |
|  |  | Depth | 0.448 | 0.127 | 0.213 | 1.164 | 0.239 | **<0.05*** | 0.716 | 0.150 | 0.141 |
| **TR** | Buccal | Constant | 3.434 |  | 0.000 | 2.926 |  | 0.000 | 1.065 |  | 0.071 |
|  |  | Angle | 0.001 | 0.008 | 0.896 | 0.001 | 0.010 | 0.882 | 0.001 | 0.016 | 0.809 |
|  |  | Depth | 0.135 | 0.047 | 0.471 | 0.062 | 0.023 | 0.724 | 0.085 | 0.033 | 0.607 |
|  | Mesial | Constant | 3.737 |  | 0.000 | 1.928 |  | 0.002 | 0.287 |  | 0.642 |
|  |  | Angle | 0.007 | 0.123 | **<0.05*** | 0.011 | 0.158 | **<0.05*** | 0.012 | 0.175 | **<0.01**** |
|  |  | Depth | 0.094 | 0.040 | 0.517 | 0.459 | 0.152 | **<0.05*** | 0.270 | 0.091 | 0.138 |
|  | Distal | Constant | 3.682 |  | 0.000 | 1.638 |  | 0.019 | 0.947 |  | 0.161 |
|  |  | Angle | 0.007 | 0.110 | 0.080 | 0.013 | 0.162 | **<0.01**** | 0.005 | 0.059 | 0.345 |
|  |  | Depth | 0.078 | 0.039 | 0.530 | 0.464 | 0.179 | **<0.01**** | 0.357 | 0.143 | **<0.05*** |

**p*<0.05, ***p*<0.01, ****p*<0.001
