# Supplementary material for: Root morphology and alveolar bone phenotype of maxillary second molars in patients with periodontitis: a cross-sectional study
Source: BMC Oral Health. 2026 Aug 19;26:1813. doi: 10.1186/s12903-026-09365-5 (PMC13613663; doi:10.1186/s12903-026-09365-5)
Supplement: Supplementary file 7 [file 12903_2026_9365_MOESM7_ESM.docx]

Table S3. Binary logistic regression analysis of BOP and root concavity.

|  |  |  | **BOP** | | |
| --- | --- | --- | --- | --- | --- |
|  |  |  | B | SE | *P* |
| **SR** | Buccal | Constant | 0.013 | 0.012 | 0.273 |
|  |  | Angle | 0.018 | 0.461 | 0.969 |
|  |  | Depth | -0.966 | 1.559 | 0.535 |
|  | Mesial | Constant | -0.009 | 0.019 | 0.624 |
|  |  | Angle | 0.098 | 1.024 | 0.924 |
|  |  | Depth | 1.584 | 2.857 | 0.579 |
|  | Distal | Constant | 0.003 | 0.011 | 0.814 |
|  |  | Angle | 0.348 | 0.522 | 0.506 |
|  |  | Depth | 0.261 | 1.745 | 0.881 |
| **DR** | Buccal | Constant | -0.012 | 0.009 | 0.191 |
|  |  | Angle | -0.474 | 0.373 | 0.204 |
|  |  | Depth | 1.661 | 1.296 | 0.200 |
|  | Mesial | Constant | 0.001 | 0.012 | 0.918 |
|  |  | Angle | 0.033 | 0.397 | 0.934 |
|  |  | Depth | 0.643 | 1.342 | 0.632 |
|  | Distal | Constant | 0.003 | 0.008 | 0.725 |
|  |  | Angle | -0.254 | 0.437 | 0.562 |
|  |  | Depth | 0.692 | 1.287 | 0.591 |
| **TR** | Buccal | Constant | 0.012 | 0.005 | 0.032 |
|  |  | Angle | 0.170 | 0.191 | 0.374 |
|  |  | Depth | -0.857 | 0.679 | 0.207 |
|  | Mesial | Constant | 0.006 | 0.005 | 0.232 |
|  |  | Angle | -0.080 | 0.200 | 0.690 |
|  |  | Depth | 0.402 | 0.686 | 0.558 |
|  | Distal | Constant | -0.005 | 0.006 | 0.411 |
|  |  | Angle | 0.108 | 0.207 | 0.602 |
|  |  | Depth | 1.215 | 0.839 | 0.148 |

**p*<0.05, ***p*<0.01, ****p*<0.001
