# Supplementary material for: Root morphology and alveolar bone phenotype of maxillary second molars in patients with periodontitis: a cross-sectional study
Source: BMC Oral Health. 2026 Aug 19;26:1813. doi: 10.1186/s12903-026-09365-5 (PMC13613663; doi:10.1186/s12903-026-09365-5)
Supplement: Supplementary file 8 [file 12903_2026_9365_MOESM8_ESM.docx]

Table S4. Multiple linear regression analysis of the root concavity angle/depth and the distal periodontal parameters of MSMs without adjacent third molars

|  |  | PD | | | CAL | | | ABL | | |
| --- | --- | --- | --- | --- | --- | --- | --- | --- | --- | --- |
|  |  | B | Standardized B | *P* | B | Standardized B | *p* | B | Standardized B | *P* |
| **SR** | Constant | 1.051 |  | 0.403 | -0.941 |  | 0.484 | -0.761 |  | 0.561 |
|  | Angle | 0.016 | 0.235 | 0.071 | 0.020 | 0.257 | **<0.05*** | 0.009 | 0.128 | 0.333 |
|  | Depth | 1.186 | 0.522 | **<0.001***** | 1.528 | 0.596 | **<0.001***** | 1.105 | 0.480 | **<0.01**** |
| **DR** | Constant | 1.786 |  | 0.210 | -0.746 |  | 0.668 | -0.192 |  | 0.923 |
|  | Angle | 0.011 | 0.199 | 0.170 | 0.014 | 0.187 | 0.180 | 0.003 | 0.034 | 0.812 |
|  | Depth | 0.878 | 0.255 | 0.080 | 1.763 | 0.402 | **<0.01**** | 1.100 | 0.231 | 0.115 |
| **TR** | Constant | 3.765 |  | **<0.001***** | 2.845 |  | **<0.01**** | 1.315 |  | 0.092 |
|  | Angle | 0.005 | 0.084 | 0.377 | 0.003 | 0.041 | 0.665 | 0.000 | 0.000 | 0.999 |
|  | Depth | 0.086 | 0.056 | 0.557 | 0.209 | 0.106 | 0.264 | 0.274 | 0.154 | 0.103 |
| *p<0.05, **p<0.01, ***p<0.001 | | | | | | | | | | |
