# Supplementary material for: Root morphology and alveolar bone phenotype of maxillary second molars in patients with periodontitis: a cross-sectional study
Source: BMC Oral Health. 2026 Aug 19;26:1813. doi: 10.1186/s12903-026-09365-5 (PMC13613663; doi:10.1186/s12903-026-09365-5)
Supplement: Supplementary file 9 [file 12903_2026_9365_MOESM9_ESM.docx]

Table S5. Multiple logistic regression analysis between the angle of root furcation, root proximity, root trunk length and FI among MSMs type.

|  | | | FI | | | | | | | | |
| --- | --- | --- | --- | --- | --- | --- | --- | --- | --- | --- | --- |
|  |  |  | 0 | | | 1 | | | 2 | | |
|  |  |  | B | Exp(B) | *P* | B | Exp(B) | *P* | B | Exp(B) | *P* |
| DR | Buccal | Y-intercept | 8.510 |  | 0.048 | 13.247 |  | 0.010 | 9.024 |  | 0.107 |
|  |  | Angle of root furcation | -0.015 | 0.985 | 0.464 | -0.035 | 0.965 | 0.178 | -0.045 | 0.956 | 0.174 |
|  |  | Root proximity | -0.777 | 0.460 | 0.234 | -1.082 | 0.339 | 0.174 | -0.865 | 0.421 | 0.338 |
|  |  | Root trunk length | -0.311 | 0.733 | 0.557 | -1.340 | 0.262 | 0.064 | -0.639 | 0.528 | 0.422 |
|  | Mesial | Y-intercept | 1.958 |  | 0.681 | / |  | / | 3.892 |  | 0.754 |
|  |  | Angle of root furcation | 0.019 | 1.019 | 0.485 | / | / | / | -0.084 | 0.920 | 0.350 |
|  |  | Root proximity | -0.745 | 0.475 | **<0.05*** | / | / | / | -0.818 | 0.441 | 0.326 |
|  |  | Root trunk length | 0.771 | 2.163 | 0.190 | / | / | / | 1.064 | 2.899 | 0.495 |
|  | Distal | Y-intercept | 6.229 |  | 0.047 | 5.294 |  | 0.117 | 7.074 |  | 0.090 |
|  |  | Angle of root furcation | 0.008 | 1.008 | 0.660 | -0.003 | 0.997 | 0.872 | -0.038 | 0.963 | 0.143 |
|  |  | Root proximity | -0.635 | 0.530 | **<0.05*** | -0.542 | 0.581 | 0.077 | -0.361 | 0.697 | 0.407 |
|  |  | Root trunk length | -0.071 | 0.931 | 0.841 | -0.076 | 0.927 | 0.846 | -0.376 | 0.687 | 0.483 |
| TR | Buccal | Y-intercept | 5.899 |  | 0.003 | 5.373 |  | 0.022 | 2.647 |  | 0.270 |
|  |  | Angle of root furcation | 0.002 | 1.002 | 0.889 | -0.015 | 0.985 | 0.373 | -0.016 | 0.984 | 0.355 |
|  |  | Root proximity | -0.477 | 0.620 | 0.071 | -0.581 | 0.559 | 0.066 | 0.063 | 1.065 | 0.842 |
|  |  | Root trunk length | -0.162 | 0.850 | 0.587 | -0.242 | 0.785 | 0.498 | -0.191 | 0.826 | 0.602 |
|  | Mesial | Y-intercept | 4.991 |  | 0.029 | -1.913 |  | 0.550 | 1.408 |  | 0.803 |
|  |  | Angle of root furcation | -0.005 | 0.995 | 0.663 | -0.005 | 0.995 | 0.730 | -0.023 | 0.977 | 0.438 |
|  |  | Root proximity | -0.247 | 0.782 | 0.197 | 0.034 | 1.035 | 0.899 | 0.085 | 1.089 | 0.862 |
|  |  | Root trunk length | 0.097 | 1.101 | 0.683 | 0.381 | 1.464 | 0.223 | -0.366 | 0.693 | 0.598 |
|  | Distal | Y-intercept | 6.309 |  | 0.007 | 5.683 |  | 0.025 | 0.749 |  | 0.809 |
|  |  | Angle of root furcation | -0.004 | 0.996 | 0.744 | -0.005 | 0.995 | 0.685 | -0.010 | 0.990 | 0.519 |
|  |  | Root proximity | -0.331 | 0.718 | **<0.05*** | -0.410 | 0.664 | **<0.05*** | -0.206 | 0.814 | 0.333 |
|  |  | Root trunk length | -0.102 | 0.903 | 0.709 | -0.111 | 0.895 | 0.709 | 0.431 | 1.540 | 0.226 |
| **p*<0.05, ***p*<0.01, ****p*<0.001 | | | | | | | | | | | |
